# Stability, Consistency and Performance of Distribution Entropy in Analysing Short Length Heart Rate Variability (HRV) Signal

**DOI:** 10.3389/fphys.2017.00720

**Published:** 2017-09-20

**Authors:** Chandan Karmakar, Radhagayathri K. Udhayakumar, Peng Li, Svetha Venkatesh, Marimuthu Palaniswami

**Affiliations:** ^1^School of Information Technology, Deakin University Melbourne, VIC, Australia; ^2^Department of Electrical and Electronics Engineering, The University of Melbourne Melbourne, VIC, Australia; ^3^School of Control Science and Engineering, Shandong University Jinan, China; ^4^Centre for Pattern Recognition and Data Analytics, Deakin University Geelong, VIC, Australia

**Keywords:** distribution entropy, heart rate variability, short-term analysis, sample entropy, approximate entropy, aging, arrhythmia

## Abstract

Distribution entropy (*DistEn*) is a recently developed measure of complexity that is used to analyse heart rate variability (HRV) data. Its calculation requires two input parameters—the embedding dimension *m*, and the number of bins *M* which replaces the tolerance parameter *r* that is used by the existing approximation entropy (*ApEn*) and sample entropy (*SampEn*) measures. The performance of *DistEn* can also be affected by the data length *N*. In our previous studies, we have analyzed stability and performance of *DistEn* with respect to one parameter (*m* or *M*) or combination of two parameters (*N* and *M*). However, impact of varying all the three input parameters on *DistEn* is not yet studied. Since DistEn is predominantly aimed at analysing short length heart rate variability (HRV) signal, it is important to comprehensively study the stability, consistency and performance of the measure using multiple case studies. In this study, we examined the impact of changing input parameters on *DistEn* for synthetic and physiological signals. We also compared the variations of *DistEn* and performance in distinguishing physiological (Elderly from Young) and pathological (Healthy from Arrhythmia) conditions with *ApEn* and *SampEn*. The results showed that *DistEn* values are minimally affected by the variations of input parameters compared to *ApEn* and *SampEn. DistEn* also showed the most consistent and the best performance in differentiating physiological and pathological conditions with various of input parameters among reported complexity measures. In conclusion, *DistEn* is found to be the best measure for analysing short length HRV time series.

## 1. Introduction

Fluctuations in RR intervals is termed heart rate variability (HRV) (Acharya et al., 2006). Parameters to quantify HRV are important diagnostic markers to determine pathological cardiac conditions (Estela et al., 1995). HRV is known to change with age, gender, disease and many such conditions (Huikuri et al., 1999; Sandercock et al., 2005). In order to quantitatively evaluate HRV, various linear methods have been proposed and shown to be effective. However, these linear techniques are not sufficient and may fail in capturing important diagnostic information since the physiological systems are highly non-linear in nature (Huikuri et al., 1999; Acharya et al., 2004). Therefore, approaches that are able to discover the non-linear dynamics within HRV are required. With such an aim, a couple of entropy measures, e.g., approximate entropy (*ApEn*) and sample entropy (*SampEn*), that reflect the complexity or irregularity of HRV have been developed (Pincus et al., 1991; Richman and Moorman, 2000; Acharya et al., 2004; Chen et al., 2009). However, these entropy measures are found to be highly parameter-dependent. Specifically, their estimations depend on data length *N*, dimension *m* (required in the delay embedding reconstruction process) and tolerance *r* (used to determine whether two vectors are similar) (Pincus, 1991; Yentes et al., 2013; Mayer et al., 2014). An incorrect choice of these parameters may lead to inconsistent results (Castiglioni and Di Rienzo, 2008; Lu et al., 2008; Liu et al., 2011; Mayer et al., 2014). Therefore, current entropy-related studies emphasize on techniques for the optimal selections of entropy parameters, thereby increasing the accuracy of complexity results (Lu et al., 2008).

Among the three parameters, the tolerance parameter *r* is considered the most critical since a small variation in the choice of *r* leads to a large difference in the assessments of complexity (Castiglioni and Di Rienzo, 2008; Lu et al., 2008; Liu et al., 2011; Mayer et al., 2014). Thus, failure to make right choice of *r* results in highly misleading results and there is no simple and reliable method for choosing the value of *r*. In an attempt to eliminate the use of *r* from entropy calculations, we have recently developed a new entropy method named distribution entropy (*DistEn*) (Li et al., 2015). Instead of binning all the vectors into similar and dissimilar categories, *DistEn* employs directly the distribution characteristics of the inter-vector distances. It introduces a bin number parameter *M* in order to estimate the empirical probability density function (*ePDF*). Unlike the tolerance *r* in *ApEn* or *SampEn* measurement, *M* is observed to be less influential on *DistEn*.

The influence of parameters *N* and *M* on *DistEn* in the case of logistic time series data is tested in previous studies and results showed that the variation of *DistEn* with *N* and *M* was negligible and thereby *DistEn* can be considered stable with respect to *N* and consistent with regard to *M* (Li et al., 2015). However, in those experiments either *N* or *M* was kept constant while varying the other. In our previous study (Udhayakumar et al., 2015), we have investigated the combined effect of *N* and *M* on *DistEn* and found that a problem-specific selection of *N* and *M* is important to achieve the best classification performance. We have also found that a random choice of *M* in *DistEn* provides better results than *ApEn* and *SampEn* in classifying arrhythmic subjects from healthy subjects (Karmakar et al., 2015) especially for short length HRV signal. However, the effect of embedding dimension *m* on the performance of *DistEn* is yet to be analysed for both synthetic and physiological time-series.

This study focuses on evaluating the combined impact of data length *N*, embedding dimension *m* and number of bins *M* on *DistEn* for both synthetic and physiological time-series. The complete *DistEn* space was revealed by varying *N*, *m* and *M* for each signal. The assessment was performed by examining the classification performance of *DistEn* as a feature for differentiating—(i) different levels of complexity in synthetic data; (ii) Young vs. Elderly, using RR interval signal; and (iii) Arrhythmia vs. Healthy, using RR interval signal. In the same context, performance of *DistEn* is compared with the earlier methods of *ApEn* and *SampEn*.

## 2. Data and methods

### 2.1. Data and subjects

Synthetic data based on logistic time-series and physiological data extracted the Physionet fantasia, MIT-BIH arrhythmia, and MIT-BIH normal sinus rhythm databases were used in this study. Data in Physionet databases are fully anonymized and thus can be used without IRB approval.

Synthetic data—Logistic time series at two different levels of complexity were used for the study. Two sets of signals with an increasing order of complexity were named as “Periodic” and “Chaotic.” In order to eliminate random factors, we generated 10 realizations (corresponding to different initial values) of the same type using the logistic map given by *x*_*n*+1_ = *ax*_*n*_(1 − *x*_*n*_). The constant *a* was set at 3.5 (or 4) with an initial value randomly chosen between 0.1 and 0.2 in order to generate a “Periodic” and “Chaotic” level signal respectively. Although larger number of realizations is better for eliminating random factors, we believe 10 realizations are well enough for this study since the domain of initial values were restricted in a small range. We only used logistic map to produce time-series with chaotic and periodic regimes since it has been the simplest and most widely used synthetic data examples to demonstrate entropy level variations (Kaplan et al., 1990; Pincus, 1991; Xie et al., 2008; Chen et al., 2009; Li et al., 2015). All synthetic signals are generated using MATLAB R2014b.

Physiologic data—RR interval data of twenty healthy “Young” (21–34 years old) and twenty healthy “Elderly” (68–85 years old) subjects were obtained from the Fantasia module of the PhysioNet database (Goldberger et al., 2000). Each data corresponds to a 120 min recording of the subject's electrocardiogram (ECG) when in continuous supine resting, sampled at a frequency of 250 Hz. Each group of subjects has an equal number of men and women. Each RR interval is computed by an automated algorithm from annotated heartbeats of subjects (Iyengar et al., 1996). After extraction of RR series of all subjects from the database, each signal segment was selected from the beginning by varying length from 50 to 1,000 beats (total 8 different lengths—50, 100, 200, 300, 400, 500, 750, and 1,000 beats corresponding to average time durations of 0.78, 1.60, 3.23, 4.88, 6.52, 8.17, 12.30, and 16.44 min, respectively) for each subject.

RR interval time-series of “Arrhythmia” and “Healthy” subjects were obtained from the MIT-BIH module of the PhysioNet database (Goldberger et al., 2000). The Arrhythmia Database contains 48 ECG recordings obtained from 47 subjects (Moody and Mark, 2001). The subjects included 25 men aged 32 to 89 years and 22 women aged 23 to 89 years. The recordings were digitized at 360 samples per second per channel with 11-bit resolution over a 10 mV range. Each beat of every record was then annotated independently using a slope sensitive QRS detector (Moody and Mark, 2001). From this, the RR interval was then computed for each subject. The Normal sinus rhythm database contains 18 long-term ECG recordings of subjects who were found to have no significant arrhythmia; they include 5 men, aged 26 to 45, and 13 women, aged 20 to 50. After extraction of RR series of all subjects from the database, each signal segment was selected from the beginning by varying length from 50 to 1,000 beats (total 8 different lengths as mentioned in previous paragraph, which corresponds to average time durations of 0.69, 1.34, 2.67, 3.98, 5.23, 6.16, 9.89, and 13.24 min, respectively) for each subject.

### 2.2. Entropy measures

In this study, we compared the characteristics and performance of *DistEn* as a entropy measure with *ApEn* and *SampEn*. The reason that *ApEn* and *SampEn* were used for comparison was because *DistEn* was initially proposed to address the dependence of the existing *ApEn* and *SampEn* methods on tolerance *r*.

#### 2.2.1. Approximate entropy (*ApEn*)

*ApEn* is an approximation of the conditional probability (Pincus, 1991; Pincus and Goldberger, 1994) of two segments matching at a length of *m*+1 if they match at *m*. The embedding dimension *m* is the length of compared segments of the input time series and *r* is the threshold of distance, which is fixed to match segments when they are compared with each other. Let a time series of length *N* be defined as {*x*(*n*) : 1 ≤ *n* ≤ *N*}. For a given value of the embedding dimension *m* and tolerance *r*, *ApEn* is calculated using following steps:

1. Form (*N* − *m* + 1) vectors of length *m* each, given by
  $$\left\{X_{i}^{m}:1\leq i\leq \left(N-m+1\right)\right\}\text{ where}$$
  (1) $$\begin{array}{l} X_{i}^{m}=\left\{x\left(i+k\right):0\leq k\leq m-1\right\} \end{array}$$
2. Take each $X_{i}^{m}$ vector of step 1 as a template vector and find its distance from every vector of $X_{j}^{m}$, where the distance is given by
  (2) $$\begin{array}{l} d_{ij}^{m}=\left\{\text{max}|X_{i}^{m}-X_{j}^{m}|:\;1\leq j\leq \left(N-m+1\right)\right\} \end{array}$$
3. Then we define
  (3) $$\begin{array}{ll} \Phi^{m}\left(r\right) & =\frac{1}{N-m+1}\sum_{\left[i=1\right]}^{N-m+1}\text{ln}C_{i}^{m}\left(r\right) \end{array}$$
  where, $C_{i}^{m}\left(r\right)$ is the probability of a vector $X_{j}^{m}$ to lie within a distance *r* of the vector $X_{i}^{m}$
4. The above steps are repeated for m+1, resulting in Φ^*m*+1^(*r*) from which *ApEn* is defined as
  (4) $$\begin{array}{l} ApEn=\Phi^{m}\left(r\right)-\Phi^{m+1}\left(r\right) \end{array}$$

In this study, we used *m* = 2, 3, 4, 5 and *r* = 0.1∗*SD* to 1∗*SD* with a step size of 0.1 ∗ *SD* to calculate *ApEn* for all signals, where SD denotes standard deviation of the signal. For ease of calculation and visualization, each RR time-series was normalized to unitary variance before calculating *ApEn*.

#### 2.2.2. Sample entropy (*SampEn*)

*SampEn* is a modified version of *ApEn* to find the irregularity of a given signal (Richman and Moorman, 2000). Here, self matches between vectors are avoided from the calculation and the same number of template vectors are used in *m* and *m*+1 dimensions. For a given time series data of length *N*, sample entropy is calculated as

(5) $$\begin{array}{l} SampEn=\ln\frac{\Phi^{m}\left(r\right)}{\Phi^{m+1}\left(r\right)} \end{array}$$

where

(6) $$\begin{array}{l} \Phi^{m}\left(r\right)=\frac{1}{N-m}\overset{N-m}{\underset{i=1}{\sum }}C_{i}^{m}\left(r\right) \end{array}$$

$C_{i}^{m}\left(r\right)$ being the probability of a vector $X_{j}^{m}$ to lie within a distance *r* of the vector $X_{i}^{m}$,1 ≤ *j* ≤ (*N* − *m*), *j* ≠ *i*.

Similar to *ApEn*, we used *m* = 2, 3, 4, 5 and *r* = 0.1 ∗ *SD* to 1 ∗ *SD* with a step size of 0.1 ∗ *SD* to calculate *SampEn* for all signals, where SD denotes standard deviation of the signal. For ease of calculation and visualization, each RR time-series was normalized to unitary variance before calculating *SampEn*.

#### 2.2.3. Distribution entropy (*DistEn*)

*DistEn* is initially developed from *SampEn* with an aim of improving the inconsistency and minimizing the dependence on input parameters. The novelty behind *DistEn* is the assumption that the inconsistency and parameter-dependence of *SampEn*-based measures come from the incomplete assessment of the distribution of inter-vector distances, and that they can be eliminated by taking full advantages of the distribution property (Li et al., 2015). By quantifying the Shannon entropy of the probability density of inter-vector distances—an assessment that completely and globally quantifies the distribution property, the so-developed *DistEn* displayed improved performance as we expected (Li et al., 2015).

For a given time series data of length *N*, embedding dimension *m* and bin number *M* the distribution entropy is calculated as follows.

1. Form (*N* − *m*) vectors of length *m* each, given by
  $$\left\{X_{i}^{m}:1\leq i\leq \left(N-m\right)\right\}\text{ where}$$
  $$X_{i}^{m}=\left\{x\left(i+k\right):0\leq k\leq m-1\right\}$$
2. Take each $X_{i}^{m}$ vector of step 1 as a template vector and find its distance from vector $X_{j}^{m}$, where the distance is given by
  $$\begin{array}{l} d_{ij}^{m}=\left\{\text{max}|X_{i}^{m}-X_{j}^{m}|:\;1\leq j\leq \left(N-m\right),j\neq i\right\} \end{array}$$
  A distance matrix *D* of size (*N* − *m*) ∗ (*N* − *m* − 1) is formed by repeating this calculation for all *i*^*th*^ template vectors, where 1 ≤ *i* ≤ (*N* − *m*).
3. The elements of distance matrix *D* are now divided into *M* number of equally spaced bins and the corresponding histogram is obtained.
4. Now, at each bin *t* of the histogram, its probability is estimated as $P_{t}=\frac{\text{count in bin }t}{\text{total number of elements in matrix }D}$; 1 ≤ *t* ≤ *M*.
5. By the definition of Shannon entropy, the normalized *DistEn* of a given time series *x*(*i*), is defined by the expression $DistEn(m,M)=\frac{-1}{\log_{2}(M)}\sum_{t=1}^{M}p_{t}\log_{2}(p_{t}),$ where *p*_*t*_ is the probability of each bin in the histogram.

In this study, we used *m* = 2, 3, 4, 5 and *M* = 50, 100, 200, 300, 400, 500, 750, 1000, 1500, 2000 to calculate *DistEn* for all signals.

### 2.3. Statistics

In our study, we used area under the ROC curve (AUC) to test the efficiency of *DistEn* as a feature to distinguish signals of different levels of complexity (synthetic) and RR time series belonging to different classes (physiologic). The AUC is the probability that a classifier ranks a randomly chosen instance *X* higher than a randomly chosen instance *Y*, *X* and *Y* being samples taken from two independent populations. An AUC value of 0.5 indicates that the distributions of the features are similar in the two groups with no discriminatory power. Conversely, an AUC value of 1.0 means that the distribution of the features of the two groups do not overlap at all. The AUC value was approximated numerically using the trapezoidal rules (Hanley and McNeil, 1982) where the larger the AUC value, the better the discriminatory performance. MATLAB R2014b Statistics toolbox was used to perform all statistical operations.

## 3. Results

The results of this study are divided into two subsections to summarize—(i) the effect of parameters on entropy values; and (ii) the performance of the entropy measurements in distinguishing various synthetic signals and physiological conditions.

### 3.1. Entropy values with varying parameters

#### 3.1.1. Synthetic signal

**Figure 1:**
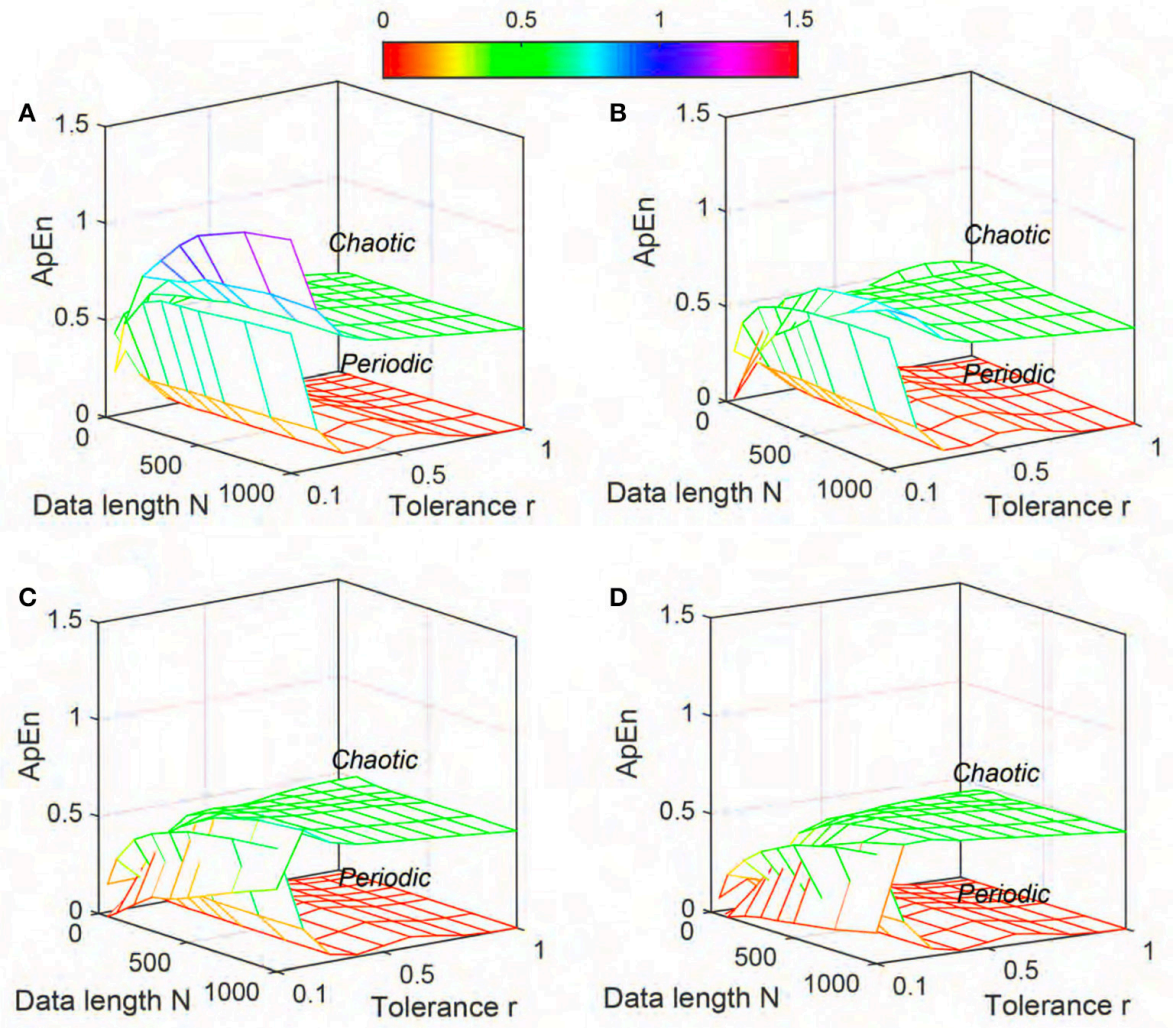
Variation of Approximate entropy (*ApEn*) for Synthetic signal (“Chaotic” and “Periodic”) varying parameters *N* and *r* for **(A)** *m* = 2, **(B)** *m* = 3, **(C)** *m* = 4, and **(D)** *m* = 5.

**Figure 2:**
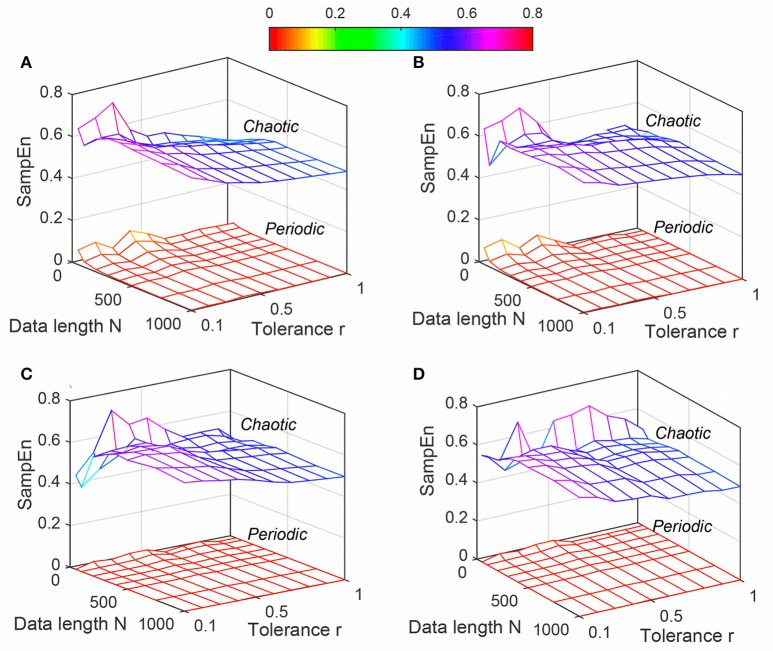
Variation of Sample entropy (*SampEn*) for Synthetic signal (“Chaotic” and “Periodic”) varying parameters *N* and *r* for **(A)** *m* = 2, **(B)** *m* = 3, **(C)** *m* = 4, and **(D)** *m* = 5.

**Figure 3:**
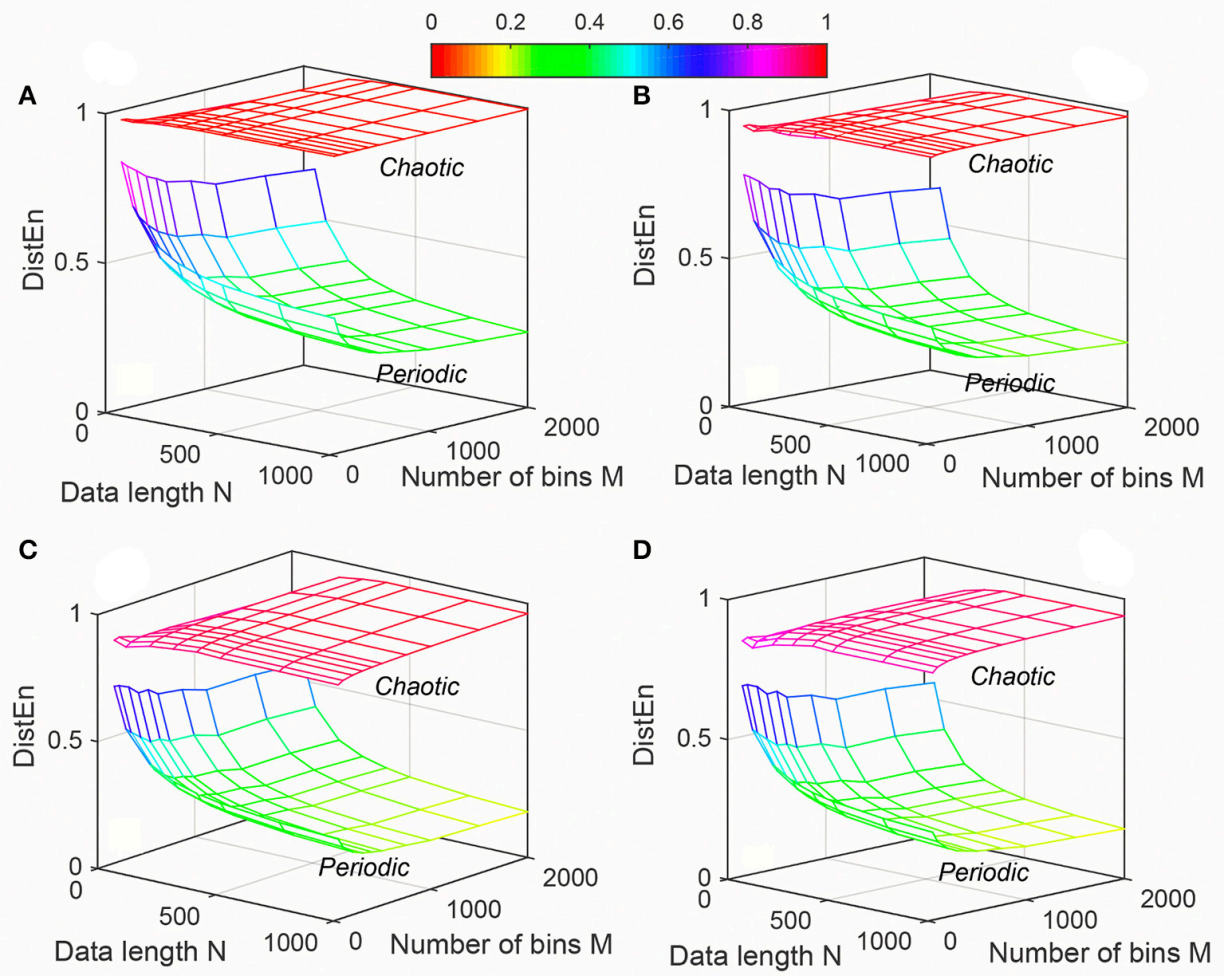
Variation of Distribution entropy (*DistEn*) for Synthetic signal (“Chaotic” and “Periodic”) varying parameters *N* and *M* for **(A)** *m* = 2, **(B)** *m* = 3, **(C)** *m* = 4 and **(D)** *m* = 5.

The variation of mean values of *ApEn*, *SampEn* and *DistEn* by varying *N*, *m* and *r* (for *ApEn* and *SampEn*) or *M* (for *DistEn*) for “Periodic” and “Chaotic” synthetic signals are shown in Figures 1–3, respectively. *ApEn* values of both “Periodic” and “Chaotic” signals were rapidly changing at low values of tolerance *r* (Figure 1). For smaller data length, such rapid variations resulted in lower mean *ApEn* value of “Chaotic” signal than “Periodic.” These characteristics of *ApEn* values remained similar for all embedding dimensions *m* = [2, 5] used in this study. Moreover, with increasing *m* values the range of data length *N* and tolerance *r* also increased for which mean *ApEn* values were unstable (Figure 1). Although average *SampEn* values varied with variation of parameters *N*, *r* and *m*, the value of “Periodic” signal always remained smaller than “Chaotic” signal (Figure 2). Moreover, the variation was more pronounced for “Chaotic” signal than “Periodic.” In contrast to *ApEn*, *SampEn* showed more variation with respect to tolerance *r* than the data length *N* for synthetic signal. In addition, similar to *ApEn*, the variation in *SampEn* values especially for “Chaotic” signal increased with increasing embedding dimension *m*. Similar to *SampEn*, average *DistEn* value was always lower in “Periodic” signal than “Chaotic” (Figure 3). However, in contrast to *SampEn* characteristics the variation in average *DistEn* values were more pronounced in “Periodic” signal than “Chaotic.” For the “Periodic” signal, *DistEn* values were affected by variations in bin number *M* for all values of data length *N*. On the other hand, for “Chaotic” signal although there was subtle variation in *DistEn* value, it was mostly due to changes in data length *N* rather than bin number *M*. Therefore, in general, the influence of *N* is more pronounced than the influence of *M* on *DistEn* of synthetic data. In addition, these characteristics of *DistEn* remained similar over all embedding dimensions *m* = [2, 5].

#### 3.1.2. Physiological signal

**Figure 4:**
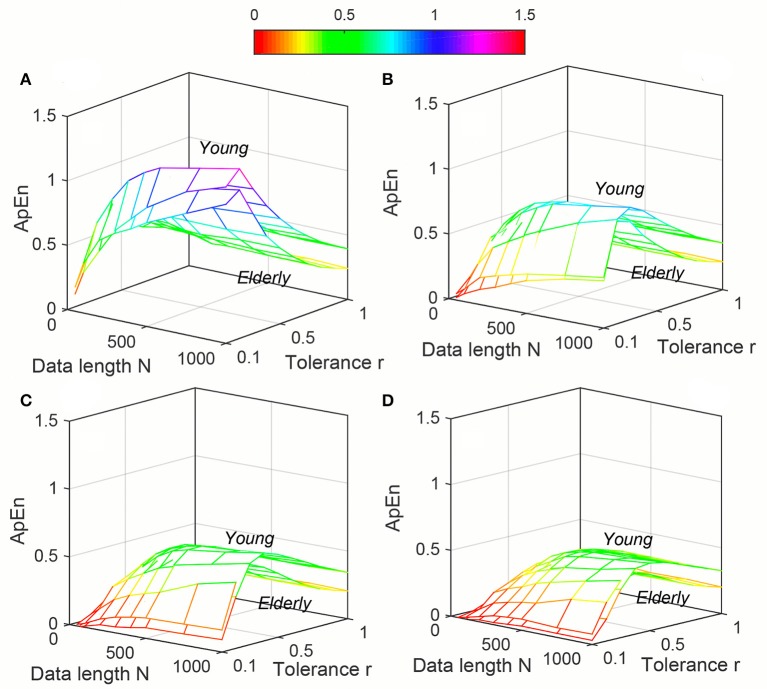
Variation of Approximate entropy (*ApEn*) for physiological signal (Elderly and Young subjects) varying parameters *N* and *r* for **(A)** *m* = 2, **(B)** *m* = 3, **(C)** *m* = 4, and **(D)** *m* = 5.

**Figure 5:**
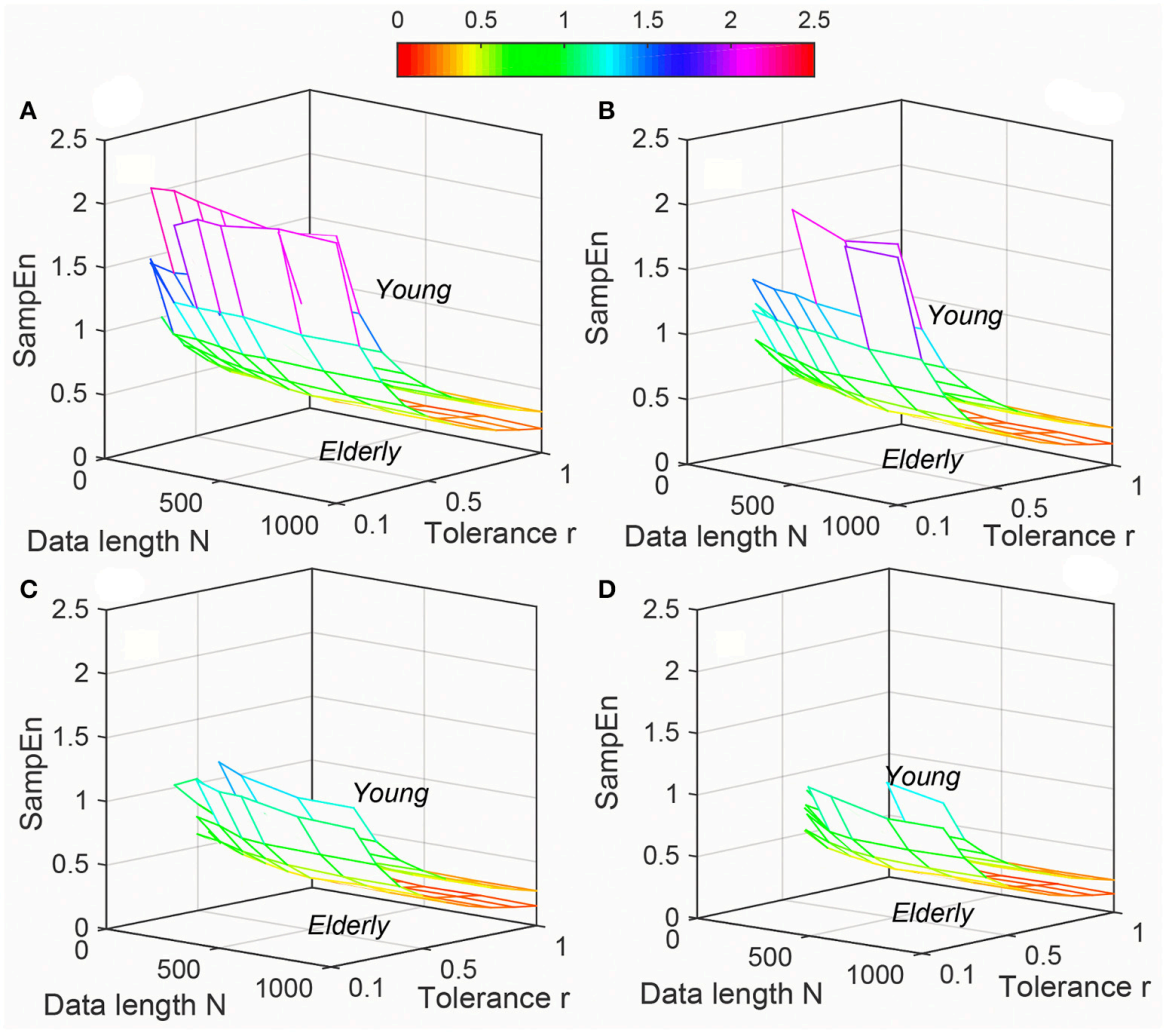
Variation of Sample entropy (*SampEn*) for physiological signal (Elderly and Young subjects) varying parameters *N* and *r* for **(A)** *m* = 2, **(B)** *m* = 3, **(C)** *m* = 4, and **(D)** *m* = 5.

**Figure 6:**
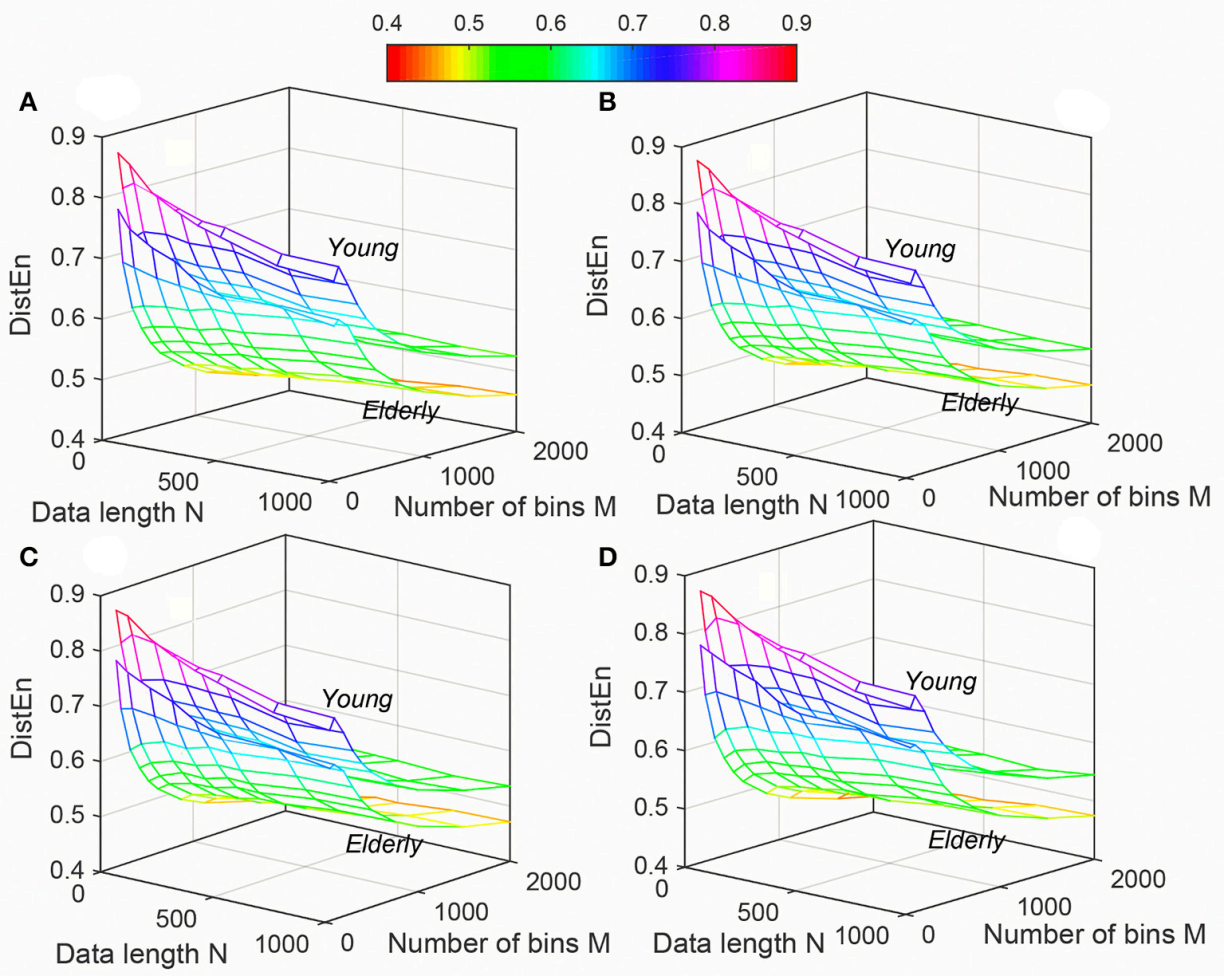
Variation of Distribution entropy (*DistEn*) for physiological signal (Elderly and Young subjects) varying parameters *N* and *M* for **(A)** *m* = 2, **(B)** *m* = 3, **(C)** *m* = 4 and **(D)** *m* = 5.

Variation of average *ApEn*, *SampEn* and *DistEn* values with varying parameter values for HRV signals of Young and Elderly population were shown in Figures 4–6, respectively. For *ApEn* and *SampEn*, effect of parameter *r* (tolerance) was predominantly higher than that of data length *N* for all embedding dimensions *m*, which was quantified as the average variance across *r* and *N* as shown in Table 1 (Columns 1 and 2 of *ApEn* and *SampEn* measures). It is obvious that $\bar{\sigma_{N}}$ is less than $\bar{\sigma_{r/m}}$ for all embedding dimensions, thus the variations of *ApEn* and *SampEn* values across different *r*-values are larger than those across different *N* values. However, in contrast to *ApEn*, *SampEn* was undefined for smaller data length either for Young or Elderly population. This undefined *SampEn* region increased with decreased *N*, decreased *r* and increased *m* (Figure 5). Table 2 (Column “Case study 1:Elderly and Young subjects”) showed the ranges of *N* and *r* that resulted in valid *SampEn* values for each embedding dimension *m*. It is obvious that with increasing *m*, higher *r* values were required for shorter data length (*N*) to obtain valid *SampEn* values. This indicates that the *SampEn* surface is sparser compared to both *ApEn* and *DistEn*, since they are both defined for all values of *N* and *r* or *M*. The variation of *DistEn* values with change of *M* was higher than variation of *N*, which indicates that the effect of bin number *M* on *DistEn* values for both Elderly and Young subjects were higher than that of *N* (Figure 6). Table 1 supported this observation quantitatively where the $\bar{\sigma_{N}}$ was lesser than $\bar{\sigma_{r/m}}$ for all embedding dimensions (see columns 1 and 2 for *DistEn* measure). This is opposite to the behavior observed for Synthetic signal and this pattern remained similar over all embedding dimensions *m* = [2, 5] (Figure 6).

**Table 1:** Average variance across data length (*N*) and tolerarance (*r* - for *ApEn* and *SampEn*) or bin number (*M* for *DistEn*) for all embedding dimension (*m*) to quantify the sensitivity of each entropy measure with respect to their parameters.

**Complexity measure**	**Embedding dimension *m***	**Young**		**Elderly**		**Healthy**		**Arrhythmia**	
		**$\bar{\sigma_{N}}$**	**$\bar{\sigma_{r/M}}$**	**$\bar{\sigma_{N}}$**	**$\bar{\sigma_{r/M}}$**	**$\bar{\sigma_{N}}$**	**$\bar{\sigma_{r/M}}$**	**$\bar{\sigma_{N}}$**	**$\bar{\sigma_{r/M}}$**
*ApEn*	2	3.11	5.91	1.70	5.88	1.58	4.55	2.60	5.38
	3	1.75	2.80	1.06	2.21	1.00	1.83	1.68	2.03
	4	1.24	2.27	0.73	1.33	0.74	1.12	1.04	1.53
	5	0.89	1.94	0.53	0.98	0.55	0.78	0.73	1.25
*SampEn*	2	0.14	27.52	0.32	24.87	3.84	15.80	0.83	23.51
	3	0.32	18.03	0.23	13.82	2.84	11.08	0.60	12.11
	4	0.31	8.51	0.33	7.57	1.62	4.38	0.47	5.09
	5	0.28	5.66	0.31	5.64	0.88	3.06	0.48	4.14
*DistEn*	2	0.04	0.98	0.03	0.81	0.08	0.48	0.05	0.38
	3	0.04	1.00	0.03	0.84	0.08	0.49	0.07	0.41
	4	0.04	1.02	0.03	0.86	0.09	0.50	0.09	0.42
	5	0.05	1.04	0.03	0.87	0.10	0.50	0.11	0.43

**Figure 7:**
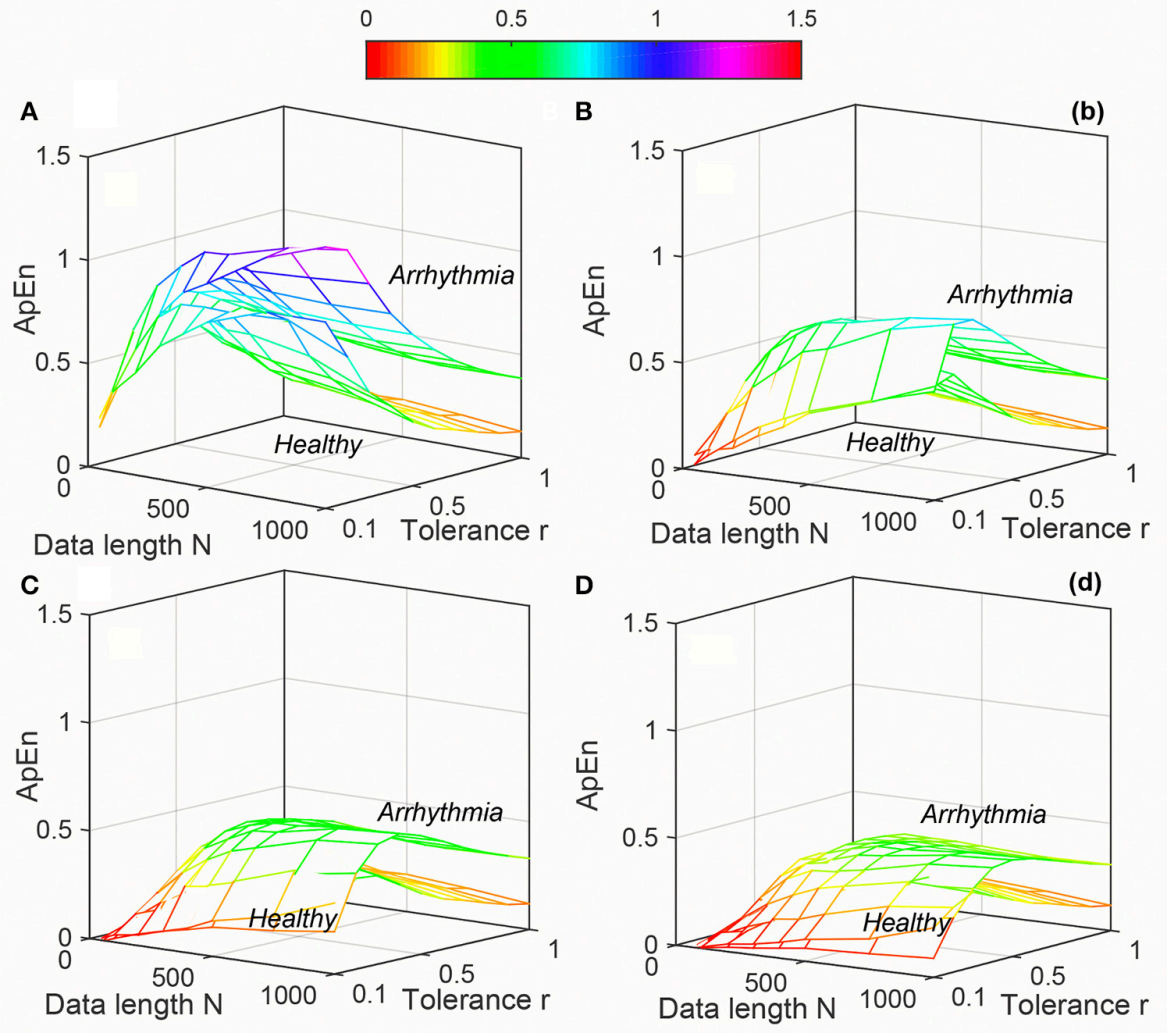
Variation of Approximate Entropy (*ApEn*) for physiological signal (Healthy and Arrhythmia) varying parameters *N* and *r* for **(A)** *m* = 2, **(B)** *m* = 3, **(C)** *m* = 4, and **(D)** *m* = 5.

**Figure 8:**
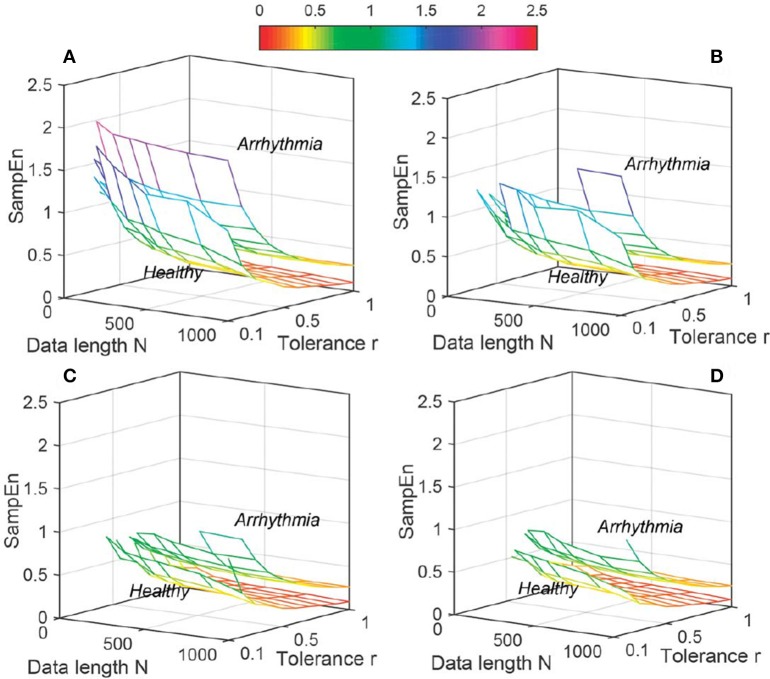
Variation of Sample Entropy (*SampEn*) for physiological signal (Healthy and Arrhythmia) varying parameters *N* and *r* for **(A)** *m* = 2, **(B)** *m* = 3, **(C)** *m* = 4, and **(D)** *m* = 5.

**Figure 9:**
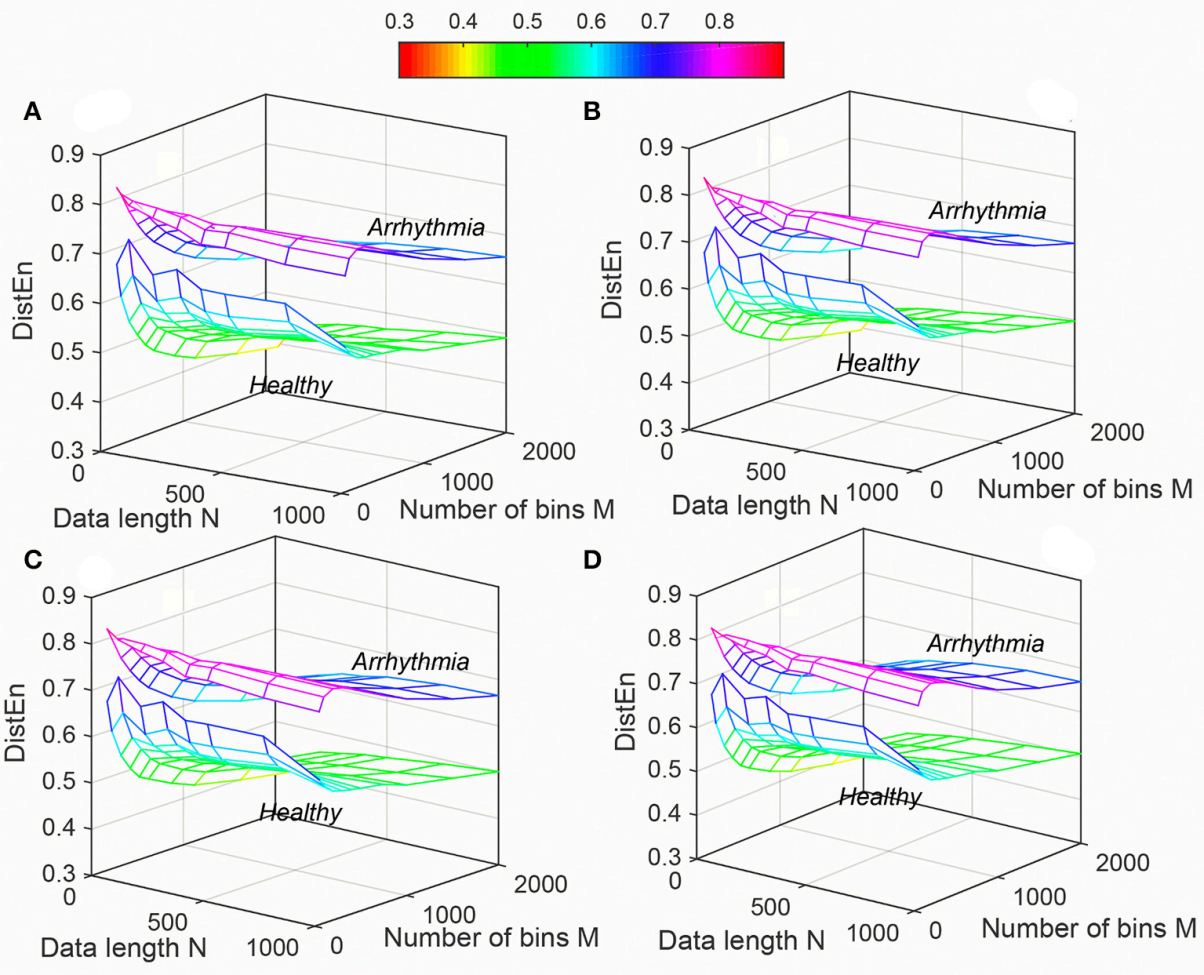
Variation of Distribution Entropy (*DistEn*) for physiological signal (Healthy and Arrhythmia) varying parameters *N* and *M* for **(A)** *m* = 2, **(B)** *m* = 3, **(C)** *m* = 4, and **(D)** *m* = 5.

*This is a quantitative summary of variations shown in Figures 4–9. $\bar{\sigma_{N}}$, Average variance measured accross data length (N); $\bar{\sigma_{r/M}}$, Average variance measured across tolerance (r) or bin numbers (M) (tolerance for ApEn and SampEn, bin number for DistEn). Values of $\bar{\sigma_{N}}$ and $\bar{\sigma_{r/M}}$ presented in this table are multiplied by 10^2^ for removing leading zeros*.

**Table 2:** Range of parameter values for which Sample entropy (*SampEn*) measure are defined for both case studies (Case study 1: Elderly and Young subjects, Case study 2: Healthy and Arrhythmia subjects).

**Embedding dimension**	**Defined range of** ***N*** **and** ***r***	
	**Case study 1: Elderly and Young subjects**	**Case study 2: Healthy and Arrhythmia subjects**
*m* = 2	*N* = 50; for 0.3 ≤ *r* ≤ 1	*N* = 50; for 0.3 ≤ *r* ≤ 1
	*N* = 100, 200; for 0.2 ≤ *r* ≤ 1	*N* = 100; for 0.2 ≤ *r* ≤ 1
	300 ≤ *N* ≤ 1,000; for 0.1 ≤ *r* ≤ 1	200 ≤ *N* ≤ 1,000; for 0.1 ≤ *r* ≤ 1
*m* = 3	*N* = 50; for 0.4 ≤ *r* ≤ 1	*N* = 100, 200; for 0.3 ≤ *r* ≤ 1
	*N* = 100; for 0.3 ≤ *r* ≤ 1	300 ≤ *N* ≤ 500; for 0.2 ≤ *r* ≤ 1
	200 ≤ *N* ≤ 500; for 0.2 ≤ *r* ≤ 1	*N* = 750, 1,000; for 0.1 ≤ *r* ≤ 1
	*N* = 750, 1,000; for 0.1 ≤ *r* ≤ 1	
*m* = 4	*N* = 50; for 0.6 ≤ *r* ≤ 1	*N* = 50; for 0.6 ≤ *r* ≤ 1
	*N* = 100; for 0.5 ≤ *r* ≤ 1	*N* = 100; for 0.4 ≤ *r* ≤ 1
	*N* = 200; for 0.4 ≤ *r* ≤ 1	*N* = 200, 300; for 0.3 ≤ *r* ≤ 1
	300 ≤ *N* ≤ 500; for 0.3 ≤ *r* ≤ 1	400 ≤ *N* ≤ 1,000; for 0.2 ≤ *r* ≤ 1
	*N* = 750, 1,000; for 0.2 ≤ *r* ≤ 1	
*m* = 5	*N* = 100; for 0.6 ≤ *r* ≤ 1	*N* = 50; for 0.7 ≤ *r* ≤ 1
	*N* = 200; for 0.4 ≤ *r* ≤ 1	*N* = 100; for 0.5 ≤ *r* ≤ 1
	300 ≤ *N* ≤ 750; for 0.3 ≤ *r* ≤ 1	*N* = 200; for 0.4 ≤ *r* ≤ 1
	*N* = 1,000; for 0.2 ≤ *r* ≤ 1	300 ≤ *N* ≤ 500; for 0.3 ≤ *r* ≤ 1
		*N* = 750, 1,000; for 0.2 ≤ *r* ≤ 1

The variation of average entropy (*ApEn*, *SampEn* and *DistEn*) values with varying parameter values for Healthy and Arrhythmia population were shown in Figures 7–9, respectively. Similar to the previous case study (Young and Elderly population), *SampEn* was the only entropy measure that was undefined for different combinations of *N*, *m* and *r*, especially at lower *N*, *r* and higher *m* (Table 2- Column “Case study 2: Healthy and Arrhythmia subjects”). In addition, variation of entropy values was higher with respect to the change of *r* than that of *m* for both *ApEn* and *SampEn* (Columns 3 and 4 of Table 1 for *ApEn* and *SampEn* measures). On the other hand, although *DistEn* values changed with varying *N* and *M*, the changes were relatively small for both of them (Table 1). Moreover, in contrast to *ApEn*, there was no crossover in *SampEn* and *DistEn* values for any combination of *N*, *m* and *r* or *M*.

### 3.2. Performance by varying parameters

Table 3 summarized the performance of the three entropy measures for classifying i) Elderly from Young and ii) Healthy (Normal Sinus Rhythm) from Arrhythmia subjects. For “Case study 1” (Elderly vs. Young) the change in average AUC was maximum for *ApEn* (0.72, 0.68) and minimum for *DistEn* (0.80, 0.79) with respect to embedding dimension *m*. Similarly, change in median AUC was also minimum for *DistEn* (0.81, 0.79) along with *SampEn* (0.75,0.73). However, the standard deviation (SD) and inter-quartile range of *DistEn* was the lowest for each embedding dimension *m* among all entropy measures. In addition, *DistEn* showed the highest average and median AUC values for each embedding dimension *m*, which indicate that *DistEn* is a better measure to distinguish Elderly from Young subjects than *SampEn* and *ApEn*. For “Case study 2” (Healthy vs. Arrhythmia), although the average AUC value of *ApEn* and *SampEn* changed with the variations of embedding dimension *m*, it remained constant for *DistEn*. Similarly, the median AUC values of *DistEn* also remained same (0.92) over the variations of embedding dimension. Interestingly, although *SampEn* showed the lowest SD of AUC values for each embedding dimension *m*, the inter-quartile range was the lowest for *DistEn*. Since performance of *SampEn* was calculated only for the range of parameters defined in Table 2, this reduced number of AUC values might lead to such small SD values. Similar to “Case Study 1,” *DistEn* was also found to be the best measure for distinguishing Healthy from Arrhythmia subjects (average and median AUC values were 0.88 and 0.92, respectively).

**Table 3:** Comparison of classification performance of *ApEn*, *SampEn* and *DistEn* for two case studies.

**Embedding dimension**	**Complexity measure**	**Elderly vs. Young**		**Healthy vs. Arrhythmia**	
		***Mean*(*SD*)**	***Median* (1st -3rd)**	***Mean*(*SD*)**	***Median* (1st -3rd)**
*m* = 2	*ApEn*	0.72(0.08)	0.74(0.68 − 0.78)	0.75(0.10)	0.77(0.68 − 0.83)
	*SampEn*	0.72(0.07)	0.73(0.68 − 0.77)	0.74(0.08)	0.77(0.71 − 0.79)
	*DistEn*	0.80(0.05)	0.81(0.76 − 0.84)	0.88(0.11)	0.92(0.89 − 0.93)
*m* = 3	*ApEn*	0.71(0.11)	0.72(0.63 − 0.80)	0.69(0.12)	0.72(0.57 − 0.79)
	*SampEn*	0.74(0.06)	0.75(0.70 − 0.80)	0.74(0.07)	0.76(0.72 − 0.79)
	*DistEn*	0.80(0.05)	0.79(0.76 − 0.84)	0.88(0.11)	0.92(0.90 − 0.93)
*m* = 4	*ApEn*	0.69(0.10)	0.70(0.61 − 0.78)	0.66(0.12)	0.66(0.56 − 0.77)
	*SampEn*	0.74(0.07)	0.74(0.68 − 0.80)	0.75(0.07)	0.77(0.73 − 0.79)
	*DistEn*	0.79(0.05)	0.80(0.76 − 0.83)	0.88(0.11)	0.92(0.90 − 0.93)
*m* = 5	*ApEn*	0.68(0.09)	0.69(0.60 − 0.76)	0.65(0.10)	0.62(0.57 − 0.74)
	*SampEn*	0.73(0.06)	0.74(0.69 − 0.80)	0.76(0.05)	0.76(0.74 − 0.78)
	*DistEn*	0.79(0.05)	0.79(0.76 − 0.82)	0.88(0.10)	0.92(0.91 − 0.93)

*Performances measured for each embedding dimension m. Mean, average AUC value; SD, standard deviation of AUC values; Median, median AUC value; 1st, AUC value at 25th percentile; and 3rd, AUC value at 75th percentile*.

Table 4 showed the best performance obtained for each entropy measure by varying parameters *m* and *r*/*M* for minimum (50 beats) and maximum (1,000 beats) data lengths. It is obvious that *DistEn* outperformed *ApEn* and *SampEn* for minimum data length (50 beats) in both case studies (AUC for *DistEn* (0.82, 0.94), ApEn (0.75, 0.67) and *SampEn* (0.75, 0.66)). In contrast to minimum data length, *DistEn* showed a comparable performance in both case studies for maximum data length (1,000 beats) (Table 4).

**Table 4:** Combination of parameters (*r*/*M,m*) that shows best classification performances (*AUC*_*max*_) of *ApEn*, *SampEn* and *DistEn* with minimum (50 beats) and maximum (1,000 beats) data lengths for two case studies used in this study.

**Data length *N***	**Complexity measure**	**Elderly vs. Young *r*/*M, m*(*AUC*_*max*_)**	**Healthy vs. Arrhythmia *r*/*M, m*(*AUC*_*max*_)**
50	*ApEn*	1, 2(0.75)	0.9, 5(0.67)
	*SampEn*	1, 2(0.75)	1, 2(0.66)
	*DistEn*	100, 4(0.82)	200, 2(0.94)
1000	*ApEn*	0.8, 3(0.85)	1, 2(0.92)
	*SampEn*	0.8, 3(0.84)	0.3, 2(0.87)
	*DistEn*	2000, 5(0.82)	400, 5(0.93)

## 4. Discussion

Several studies have shown that HRV is capable of tracking cardiovascular disease development (Thayer et al., 2010), assessing mental disorders (Kemp and Quintana, 2013), and reflecting autonomic dysregulation (Sgoifo et al., 2015). The recent emergence of wearable devices and mobile applications further promotes the development of this translational field by offering the opportunity for continuous and long-time monitoring of HRV (Walsh et al., 2014). To achieve this goal, the quantification methods should be able to accept short or even extremely short HRV series as input without (or minimally) affecting the results.

Distribution entropy (*DistEn*) has been shown to be a reliable measure of complexity for short length HRV time series (Li et al., 2015; Udhayakumar et al., 2015). *DistEn* takes full advantage of the state space representation of the original HRV series, by measuring the probability distribution from all inter-vector distances, to alleviate the problem that traditional measures, e.g., approximate entropy (*ApEn*) and sample entropy (*SampEn*), suffer for short length signal (Li et al., 2015). The fact that time series with different dynamics have different distribution profiles, suggests the distribution property probably intrinsic and provides a rationale for *DistEn* to employ the probability density function of the distances as a media for complexity analysis (Li et al., 2015). Performance of DistEn has been tested by surrogate data analysis, simulation models, and real experimental data (Li et al., 2015; Udhayakumar et al., 2015). *DistEn* introduced another parameter—*M* (bin number used to estimate the empirical probability density) to replace *r* (threshold value)—in *ApEn* and *SampEn* calculation. We have shown using both benchmark data and real HRV series that the selection of *M* is not as critical as *r* (Li et al., 2015; Udhayakumar et al., 2015). We have also proved that *DistEn* remains relatively stable with extremely short series whereas the two traditional measures fail. In addition to *M* and *N*, there is yet another parameter—*m* (embedding dimension)—that needs to be considered in order to fully span the *DistEn* space. Together, they may have some combined impacts on *DistEn* performance, which has not yet been determined and that motivated our current study.

Intriguingly, the results are as what we expected:

- *DistEn* varied less with different combinations of *m*, *M*, and *N* as compared with *ApEn* and *SampEn* (Figures 3, 6, 9).
- For even very small data lengths, *N*, *DistEn* still could result in reasonable values rather than invalid or extreme values (Table 2).
- *DistEn* performed the best among the three in differentiating Elderly subjects from Young, or differentiating Arrhythmia subjects from Healthy (Table 3).
- Performance of *DistEn* is minimally affected by the input parameters compared to *ApEn* and *SampEn* in both case studies (Table 3).
- The best performance of *DistEn* is always the highest among the three entropy parameters in differentiating Elderly subjects from Young, or differentiating Arrhythmia subjects from Healthy for shortest data length and comparable for longest data length (Table 4).

In brief, the main findings of this study proved the stability and consistency of *DistEn* with variations of input parameters. *DistEn* also showed better performance in distinguishing healthy “Young” from “Elderly” and “Healthy” from “Arrhythmic” subjects than that with other popular entropy measures *ApEn* and *SampEn*. The results of this study showed that average AUC of *DistEn* varied least with change of embedding dimension (compared to *ApEn* and *SampEn*). Also, the average AUC of *DistEn* remained the highest among those of the studied entropy measures. This indicates that *DistEn* is the best feature and the performance is minimally affected by the choice of entropy measurement parameters. In addition, low inter-quartile range (IQR) of AUC value of *DistEn* further establishes the findings that the performance of *DistEn* is least affected by choice of parameters compared to *ApEn* and *SampEn*.

From results, it is obvious that *DistEn* showed the best performance (maximum AUC value for any combination of parameters) for minimum data lengths (Table 4). Although the differences in margins among the best performances for “Case study 1” are small, these performances of *ApEn* and *SampEn* are obtained with very high tolerance *r* values (0.9 and 1), which fall outside the range of traditionally recommended range (between 0.1 and 0.25). On the other hand, for longest data length (1,000 beats) the best performance of *DistEn* is comparable with *ApEn* and *SampEn*. This further suggests that existing entropy measures fail in short-length data, although they show good performance for long-length data. However, similar to short length data, most of these performances of *ApEn* and *SampEn* are obtained at very large tolerance values (0.8 and 1) except “Case study 2” of *SampEn*, where tolerance value (*r* = 0.3) remains within the traditional range. These results further prove that performance of *ApEn* and *SampEn* is highly sensitive and inconsistent with varying parameters compared to *DistEn*.

Findings of this study shows that higher tolerance (*r*) values are needed for obtaining *SampEn* values for short length signal, which is aligned with previously reported findings (Xie et al., 2008; Li et al., 2015). The minimum *r* value for obtaining *SampEn* value also increases with increasing value of embedding dimension *m* (Table 2). The *SampEn* measure becomes undefined for tolerance values for which none of the vector matches with any of the template vectors i.e., ϕ^*m*^(*r*) = 0 or ϕ^*m*+1^(*r*) = 0. This indicates that lenient tolerance value is necessary for measuring *SampEn* of short length signal. In contrast to *SampEn*, both *ApEn* and *DistEn* remain defined for all data lengths. The *ApEn* measure is defined for any data length and tolerance value, since it considers self-matching of template vectors i.e., the probability of a vector to lie with in a distance *r* of the template vector is always greater than zero ($C_{i}^{m}\left(r\right)>0$ or $C_{i}^{m+1}\left(r\right)>0$). On the other hand, since *DistEn* measure uses all distances between each pair of vectors in state-space to generate the probability distribution, it is always defined for any data length.

In the case of Synthetic signals, although subtle variations were present in the values, all three measures used in this study (*ApEn*, *SampEn*, and *DistEn*) were able to perfectly distinguish (*AUC* = 1) “Chaotic” signal from “Periodic” signal for all combination of parameters. However, such consistency in performance was not found for physiological signal where the signal is neither periodic nor chaotic. In particular, for short length signal the performance of *ApEn* and *SampEn* was worse compared to *DistEn*. However, both *ApEn* and *SampEn* showed better or comparable performance with *DistEn* in case of larger data length (*N* = 1,000). This indicates that the consistency and performance of *ApEn* and *SampEn* are highly affected by the choice of parameters especially for physiological signal analysis.

The calculation over the inter-vector distances in *DistEn* algorithm may essentially account for its improvement. Specifically, by estimating the probability density of all inter-vector distances, the amount of used information in *DistEn* is strikingly increased from the order of *N* to *N*^2^. When *N* is small, the estimation of the probability of only “similar vectors” in *ApEn* and *SampEn* will become severely unreliable (due to inadequate information of the “similar vectors” though *ApEn* and *SampEn* indeed also calculate all the inter-vector distances), whereas *DistEn* will not be affected significantly with the increased information. In a previous study, we also proved that the use of the probability density rather than the probability of only “similar vectors” is theoretically reasonable (Li et al., 2015). With this study, we further confirmed our previous hypothesis that the performance of complexity estimates could be improved by globally quantifying the inter-vector distances in the state-space (Li et al., 2015).

Given the methodological discrepancies between *DistEn* and compared measures (*ApEn* and *SampEn*), it is reasonable to recall the existing questions—what is complexity and how can it be measured. Although until most recently, they remain being the source of many scientific arguments (Mitchell, 2009), researchers have proposed some measurements to estimate complexity from different scenarios, the irregularity of time-series which can be quantified by *ApEn* and *SampEn* included. However, a high irregularity level may not necessarily be indicative of a high complexity; instead, the irregularity increases with the degree of randomness (Costa et al., 2002). In our previous work (Li et al., 2015), we tried to propose a concept that the “spatial structure”, instead of only the conditional probability of similar vectors that have been used in *ApEn* and *SampEn*, might be an indicative feature of complexity. We applied the empirical probability density function (*ePDF*) of inter-vector distances to characterize this spatial structure in *DistEn* measure and found that time-series with different dynamics had distinctly different *ePDF*s: (i) a time-series with chaotic regime is accompanied by dispersedly distributed inter-vector distances; and (ii) the distribution becomes concentrative for periodic time-series. Based on this concept, periodic time-series are not always accompanied with a *DistEn* of 0 since they could indicate different distribution patterns in term of the spatial structure and consequently, it could be reasonable to offer them different complexity levels. The correlation of *DistEn* with Lyapunov exponent (*LE*) for a series of logistic maps has been studied to show that *DistEn* correlates with *LE* or KS entropy in a chaotic system. We have also studied the correlation between *DistEn* and *SampEn*. The results shows that the correlation between *DistEn* and *LE* is higher than *DistEn* and *SampEn* (Figure S3 in Supplementary Material). In addition, since *DistEn* was initially developed with the aim of solving the parameter- and length-dependence of *ApEn* and *SampEn*, we restricted the comparisons to only *ApEn* and *SampEn* in the current study. However, other measures, e.g., Lyapunov exponent, fractal dimension, recurrent quantification analysis based measures, and moment statistics, may also potentially be comparative algorithms. Although the study reported synthetic signal generated using logistic map with chaotic and periodic regimes, performance of *DistEn* in distinguish other chaotic and random behaviors are shown in Figures S1, S2 (Supplementary Material). Finally, it should be noted that the method we have applied to assess the distribution property—the estimation of empirical probability density—is exactly the first trial in order to prove our initial assumption, that is, complexity of time series can be more robustly assessed by taking full advantages of the distribution property of inter-vector distances (Li et al., 2015). The current study has given us chance to understand more about the performance of *DistEn* and its possible limitations, e.g., how estimation of empirical probability density affects the outcome. Further exploration in this regard should be warranted to improve the *DistEn* measurement.

The complexity or irregularity in HRV is a long established bio-marker to evaluate health status (Lipsitz and Goldberger, 1992) and the recent studies with HRV measurement has shown the capacity of predicting the future health status using it (Van Gestel et al., 2011; Hsiung et al., 2015). This study analyses the comprehensive characteristics of different entropy measures for short-length HRV series, which will be of significant help in selecting appropriate entropy measure and its parameters for future applications.

## Author contributions

CK, RU, and PL contributed to the conceptualization, data analysis and interpretation of results. CK drafted the manuscript. PL, SV, and MP critically revised the significant intellectual content of the work. All authors approved the final version of the manuscript.

### Conflict of interest statement

The authors declare that the research was conducted in the absence of any commercial or financial relationships that could be construed as a potential conflict of interest.
