# Supplementary material for: Stability, Consistency and Performance of Distribution Entropy in Analysing Short Length Heart Rate Variability (HRV) Signal
Source: Front Physiol. 2017 Sep 20;8:720. doi: 10.3389/fphys.2017.00720 (PMC5611446; doi:10.3389/fphys.2017.00720)
Supplement: Supplementary file 1 [file Image1.PDF]

Supplementary Material

Stability, Consistency and Performance of Distribution Entropy in Analysing Short Length Heart Rate Variability (HRV) signal

Chandan Karmakar*, Radhagayathri K. Udhayakumar, Peng Li*, Svetha Venkatesh, Marimuthu Palaniswami

*** Correspondence:**

Chandan Karmakar: karmakar@deakin.edu.au

Peng Li: pli@sdu.edu.cn

1. Some systematic tests of DistEn as a measure of complexity

In order to better understand the variations of DistEn among different dynamics, e.g., how DistEn differs between chaotic and random behaviors, we re-performed the simulation experiments as described in [1]. In the simulation experiments, five synthetic signals were used: i) logistic chaotic series generated by the logistic map $x(n + 1) = \mu \times x(n) \times (1 - x(n))$ with $\mu = 4.0$; ii) logistic periodic signal generated with $\mu = 3.5$; iii) MIX(0.1) process (a MIX(p) process is in nature sinusoid signal of length N, where $N \times p$ randomly chosen points are replaced with independent identically distributed random noise); iv) MIX(0.2) process; and v) Gaussian white noise.

Two simulation tests were done:

1) To assess the algorithms' sensitivity to data length, we evaluated DistEn in the aforementioned five series as a function of data length N, which was set at ten different values from 50 to 2,000 logarithmically. We chose $m = 2$ and $M = 512$ in all calculations of DistEn. Similarly, we also calculated the ApEn and SampEn results with parameters $m = 2$ and $r = 0.2\sigma$ (where σ is the standard deviation) for comparison purposes.

2) The DistEn is a function of m and M. Actually, M serves as an intermediate parameter just as what r plays in ApEn and SampEn. We have mentioned that traditional ApEn and SampEn measures lack consistency because they are all extremely sensitive to r. Then, we should here first show the dependence of DistEn on M. We set M at 40 different values chosen from 128 (2^7) to 1,024 (2^{10}) with equal steps. For comparison purposes, we calculated the ApEn and SampEn results with 20 r values chosen from 0.025 to 0.5 with a step of 0.025.

Results for these two simulation tests are shown in Figs. S1 and S2, respectively.

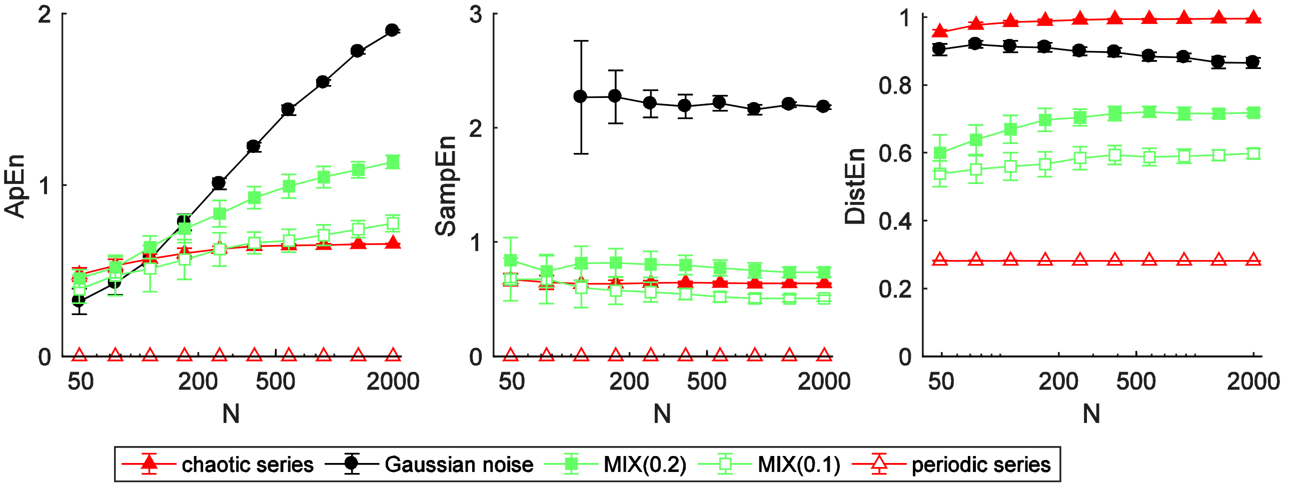

Figure S1. ApEn, SampEn, and DistEn of 5 series are shown as functions of data length. Error bar indicates the standard deviation of 20 realizations. The abscissa is shown in logarithmic scale. Note that the error bar of DistEn is very trivial.

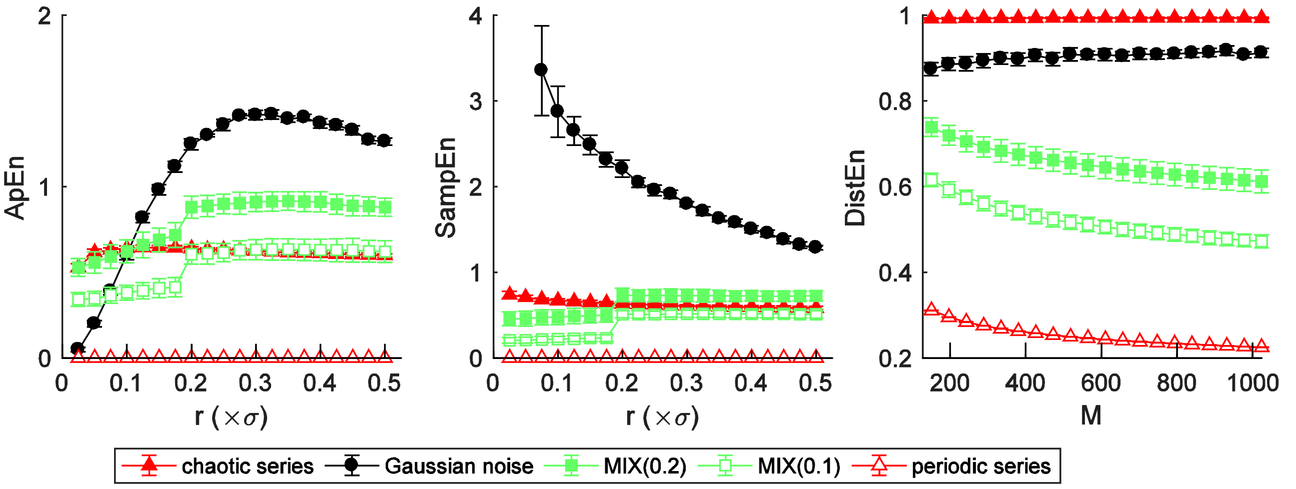

Figure S2. ApEn, SampEn of 5 series are shown as functions of *r* and DistEn as a function of *M*. Error bar indicates the standard deviation of 20 realizations.

2. Correlation of DistEn and SampEn with Lyapunov Exponent

Unlike SampEn, the calculation of DistEn is not conditional entropy-based even though the original idea of DistEn is proposed based on detailed dissection of SampEn [1]. The relation of DistEn with other complexity measures may also potentially be interesting. Here we explored the correlation between DistEn and Lyapunov exponent (LE) and the results are shown in Figure S3. As a comparison, we also showed the correlation of DistEn and SampEn in Figure S3. The average correlation between DistEn and LE is higher than the correlation between SampEn. The simulated signal was generated by varying μ from 3.5 to 4.0 for logistic map $x(n + 1) = \mu \times x(n) \times (1 - x(n))$. Length of simulated signal was always 500 and in order to stabilize the dynamics, we generated 1,000 points and removed the first 500 points. A total of 20 initial values selected randomly from 0 to 1 were used. All SampEn values were calculated for $m = 2$, $r = 0.2$ and bin number 256 was used for calculating all DistEn values. The top panels in Fig. S3 show DistEn and LE as functions of μ (top left), and DistEn and SampEn as functions of μ (top right). Correlation was performed between each pair of profiles (i.e., profile of DistEn vs. profile of LE, and profile of DistEn vs. profile of SampEn) with the same initial value. For each profile, 1000 instances of simulated signal were generated by varying μ from 3.5 to 4.0 for logistic map to compute entropy values and their correlation. Results for correlation analysis were presented in the bottom two panels in Fig. S3.

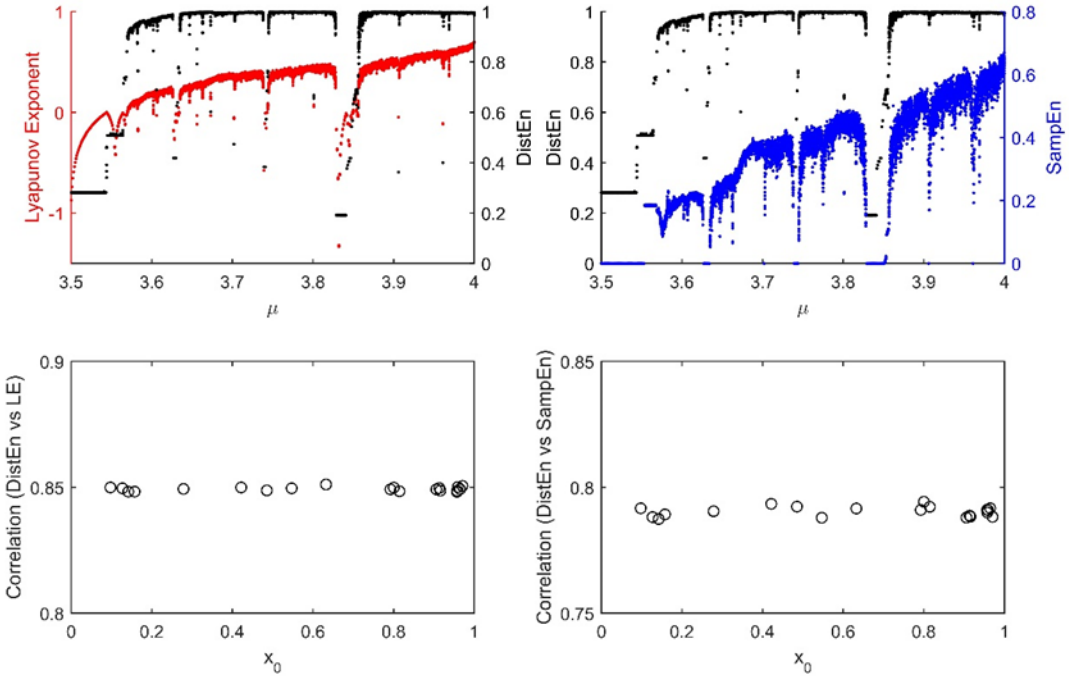

Figure S3. DistEn ($m = 2$, bin number =256), Lyapunov exponent (LE) and SampEn ($m = 2$, $r = 0.2$) for logistic map with μ values from 3.5 to 4.0 and length of 500.

References:

[1] Li, P., Liu, C., Li, K., Zheng, D., Liu, C., and Hou, Y. (2015). Assessing the complexity of short-term heartbeat interval series by distribution entropy. Medical & Biological Engineering & Computing, 53(3): 77-87.
